# Bowel preparation assessment using artificial intelligence: Systematic review

**DOI:** 10.1055/a-2625-6327

**Published:** 2025-07-01

**Authors:** Kristoffer Mazanti Cold, Amaan Ali, Lars Konge, Flemming Bjerrum, Laurence Lovat, Omer Ahmad

**Affiliations:** 1 615551 Rigshospitalet, CAMES, Copenhagen, Denmark; 2 4919 Wellcome/EPSRC Centre for Interventional & Surgical Sciences (WEISS), University College London, London, United Kingdom of Great Britain and Northern Ireland; 3 Centre for Clinical Education, University of Copenhagen and the Capital Region of Denmark, Copenhagen, Denmark; 4 Division of Surgery & Interventional Science, University College London, London, United Kingdom of Great Britain and Northern Ireland

**Keywords:** Endoscopy Lower GI Tract, Polyps / adenomas / ..., CRC screening, Quality and logistical aspects, Preparation, Training, Performance and complications

## Abstract

**Background and study aims:**

Insufficient bowel preparation is the leading cause of missed adenomas in colonoscopy. The Boston Bowel Preparation Scale (BBPS) is the most thoroughly validated and widely used scale to estimate risk of missed adenomas. Artificial intelligence (AI) could automatically quantify bowel preparation, thus reducing bias and limitations inherent in human rating. This systematic review aimed to identify, describe, and evaluate all AI-BPS systems for colonoscopy.

**Methods:**

A systematic literature review was conducted using MEDLINE, EMBASE, and SCOPUS based on three sets of terms aligned with the inclusion criteria: colonoscopy, BPS, and AI. Two reviewers independently evaluated and completed data extraction from the articles.

**Results:**

A total of 1,449 studies were identified, with eight meeting the eligibility criteria. Six AI-BPS systems were trained on expert BBPS ratings, and two studies used a fecal-mucosal ratio. All studies compared their AI-BPS with expert BBPS ratings; two showed that their AI-BPS outperformed expert BBPS ratings, and six showed comparable performances. Three studies also demonstrated correlations with adenoma detection rates (ADRs), adenoma miss rates (AMRs), or polyp detection rates (PDRs). Only one prospective study implemented its AI-BPS, finding lower AMR in adequately prepared compared with inadequately prepared bowels.

**Conclusions:**

AI-BPS can standardize and outperform human bowel preparation evaluation by better correlating with expert BBPS ratings, AMR, ADR, and PDR. Further research following recommended reporting guidelines is needed to allow for cross-study comparisons and meta-analysis, which was not possible in this study due to heterogonous study design and reporting metrics.

## Introduction

The volume of colonoscopies is steadily increasing with evidence-based implementation of screening programs, which has reduced colorectal cancer mortality 1 . Likelihood of interval cancer correlates with quality of colonoscopy 2 , and adequate inspection of the colonic mucosa relies on sufficient bowel preparation 3 . Insufficient bowel preparation can result in premature termination of colonoscopy, thereby increasing healthcare costs and exacerbating endoscopy service pressures through need for repeat examinations 4 . Although borderline bowel preparation frequently allows the procedure to be completed, missed pathology is a grave concern 3 , with 35% to 42% of missed adenomas in colonoscopy being due to insufficient bowel preparation 5 6 . The importance of adequate bowel preparation as the leading cause of missed adenomas has garnered more attention in the world of endoscopy as a critical factor for which optimization could translate to improved outcomes.

Innovative technologies like applications based on artificial intelligence (AI) are coming to the fore to tackle this issue. Such innovations include cell phone applications assessing adequate bowel preparation by asking patients to photograph their stool pre-procedure, improving quality of bowel preparation 7 . Currently, accurate assessment of sufficient bowel preparation during the procedure relies solely on assessment by the endoscopist, which can be subjective. Several endoscopic-based scoring systems exist to ensure standardization of evaluation of bowel preparation 8 . The Boston Bowel Preparation Scale (BBPS) is the most thoroughly tested and widely used 8 . Initial studies exploring the validity of BBPS found near-perfect interrater variability 9 10 . However, two major quality analysis programs based on > 35,000 colonoscopies each questioned its applicability, because they found no correlation between adenoma detection rate (ADR) and BBPS 11 12 . With evolving use of AI, which has been applied to improve bowel preparation in the pre-procedure phase, AI could also provide a more refined and objective measure of bowel preparation quality than human-rated BBPS during the procedure. Similar AI-based systems have already proven their efficacy when used during colonoscopy. Computer-aided detection (CADe) augments endoscopist ADRs 13 . Computer-aided quality assurance (CAQ) can also help endoscopists with quality assurance with use of a withdrawal speedometer, improving mucosa inspection quality 14 . Systematic reviews and meta-analyses in CADe and CAQ have shown improved performance through a higher ADR when using these AI systems 13 14 . However, no systematic review has yet focused on use of AI in another leading cause of missed adenomas 15 16 : bowel preparation quality.

This systematic review aimed to identify, describe, and evaluate AI-BPS systems for colonoscopy compared with human ratings.

## Methods

The systematic review adhered to the Preferred Reporting Items for Systematic Reviews and Meta-Analyses (PRISMA) guidelines 17 ( **Supplementary Material 1** ). The review was conducted by searching MEDLINE (via PubMed), Embase, and SCOPUS. The primary investigator (KC) made the search string with the assistance of a research librarian from the University of Copenhagen. The search string was crafted using Boolean operators and composed of the three key elements that constitute the eligibility criteria for PubMed. The search string was then adapted for the other databases ( **Supplementary Material 2** and **Supplementary Fig. 1** ).

All articles were imported to Covidence systematic review software (Veritas Health Innovation, Melbourne, Australia), which automatically removed duplicates. The first and second author (KC and AA) independently screened all remaining articles, first by title and abstract and second by full text. Any discrepancies were resolved through consensus between the two screening authors. If consensus could not be reached, the last author, OA, made the final decision.

### Eligibility criteria

All original studies developing or investigating an AI-BPS system for individual colonoscopy procedures in a clinical setting were included. To be included, the articles should meet all the following eligibility criteria, which constituted the three key elements of the search string ( **Supplementary Material 2** and **Supplementary Fig. 1** ).

Inclusion criteria: The AI-BPS system should be able to evaluate bowel preparation of a colonoscopy video recording in a clinical setting.

The AI-BPS assessment tool should be automated and instantly be able to calculate a bowel preparation score using AI. AI is broadly defined as machines that emulate human cognitive behavior 18 based on a training dataset. We included multiple definitions of AI (machine learning, neural network, deep learning, etc.) that rely on automated image recognition to be considered AI and included in the search string ( **Supplementary Material 2** and **Supplementary Fig. 1** ).

Exclusion criteria: The BPS system could only evaluate still images or bowel preparation in simulated (non-human) environments.

Conference abstracts: Articles not in English

In addition, forward- and backward citation searching were employed, meaning that articles citing the included studies and the references in the included studies were assessed for eligibility through webofscience.com (Clarivate, Philadelphia, Pennsylvania, United States) and imported into Covidence.

### Data extraction

Data extracted from the included studies was divided into the following parts:

1. General study characteristics ( Table 1 ): Included year of publication, journal with impact factor, sampling institutions, primary and secondary outcome measures, and comparison to human ratings, among others.
2. Development and performance of the AI-BPS systems ( Table 2 ): The method used to train the AI along with the data sources on which it was trained and validated.
3. Performance metrics of the AI ( Table 3 ): Included accuracy, sensitivity, specificity, area under the receiver operating curve (AUC), negative predictive value (NPV), and positive predictive value (PPV) for internal and external validation.
4. Quality Assessment ( Table 4 ): Both reviewers assessed methodological quality using the Medical Education Research Quality Instrument (MERSQI), an 18-point scale 27 . We used this instrument because BPS is an assessment of bowel preparation. MERSQI includes Messick’s validity framework for gathering validity evidence for assessments, which has been used for quality assessment in a comparable systematic review of CAQ systems 14 and is recommended by The American Educational Research Association when evaluating validity 28 29 30 . Therefore, MERSQI was chosen to evaluate methodological quality.

For descriptive statistics provided in the tables, means and range was used.

## Results

### Study selection

**Fig. 1:**
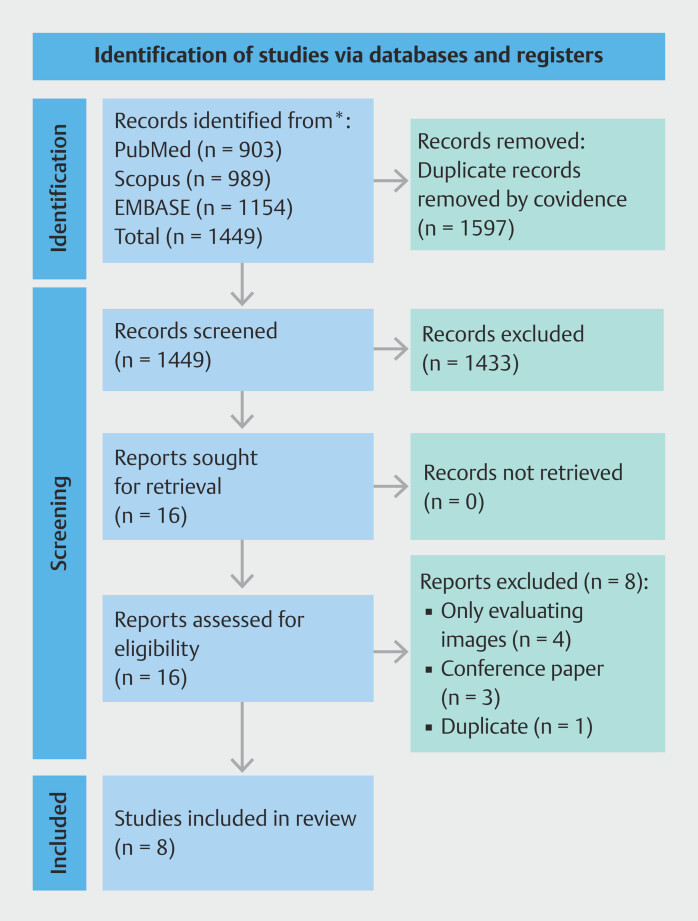
PRISMA flow chart 31 . The searches were conducted on August 15, 2024 and November 21, 2024. The chart represents the joint searches.

Searches were conducted on August 15, 2024 and November 21, 2024, identifying 1,449 studies, with eight passing the eligibility criteria ( Fig. 1 ) 19 20 21 22 23 24 25 26 .

### General study characteristics

Studies were published from 2015 to 2024 with four from China, one from South Korea, one from the United States, one from Italy, and one from Denmark ( Table 1 ). Indications for colonoscopy varied: Five studies used all colonoscopy recordings from their institution regardless of indication, one used screening colonoscopies, one used FIT-positive colonoscopies, and one did not report on indication for colonoscopy. Four studies used experienced endoscopists, one study used endoscopists of varying experience levels, and three studies did not report on endoscopist characteristics. All studies compared their AI-BPS with expert BBPS ratings. Three studies did so by using Fleiss Kappa, two using accuracy, one using interclass correlation coefficient, one using Chi square test, and one using Pearson’s correlation coefficient. Thereby, only three studies (two accuracy and one Chi square test study) were able to compare whether AI was superior to expert rating in BBPS scoring, because the five other studies only tested for correlations between the two. Unfortunately, the inconsistency index ( *I ^2^* ) could not be calculated because none of the three studies (two accuracy and one Chi square test study) reported effect sizes or their variances comparing AI and expert ratings. Consequently, a meta-analysis was not feasible due to the lack of necessary data for synthesis and heterogeneous study designs.

**Table:** **Table 1** General study characteristics of AI-BPS systems.

Study	Publication year	Country (patients)	Hospitals (sampling institutions)	Patients (population, amount)	Endoscopists performing the procedure	Primary outcome	Secondary Outcome	Study conclusion of comparison of AI to expert BBPS rating	Reporting used for Comparison between AI and expert BBPS rating
Rosa-Rizzotto et al. 19	2015	Italy	4	Screening, 50 colonoscopies	7 skilled endoscopists	Agreement between AI and expert rating	Improvement pre and post lavage	Equal	Interclass correlation coefficient = .77
Zhou et al. 20	2020	China	1	Not reported, > 2000 colonoscopies	Not reported	Agreement between AI and expert rating on images	Agreement to Expert rating on videos	Superior	Accuracy: 93.3% vs. 75.11%, *P* = 0.92
Zhou et al. 21	2021	China	1	Age 18–75 years, 616 colonoscopies	4 endoscopists with at least 5 years of experience	ADR	PDR, and agreement to expert rating on videos	Superior	Chi-square test: 10.22% vs. 9.33%, *P* = 0.08
Lee et al. 22	2022	USA	1	All colonoscopies, 200 colonoscopies	Not reported	AUC	Agreement between AI and expert rating	Equal	Fleiss’ kappa = .649
Feng et al. 23	2023	China	5	All sorts of colonoscopies, 200 colonoscopies	10 endoscopists of varying experience level	AUC	Sensitivity, specificity, and accuracy compared to experts' rating	Equal	Accuracy: 93.8% vs 90.6%, *P* > 0.05
Lee et al. 24	2024	South Korea	1	All colonoscopies, 693 colonoscopies	Not reported	Agreement between AI and expert rating	NA	Equal	Fleiss’ kappa = 0.686
Yao et al. 25	2024	China	1	Screening colonoscopies, 393 colonoscopies	3 expert endoscopists	AMR (> 5 mm)	PMR, PDR, ADR, AMR < 5 mm), Agreement between AI and expert rating	Equal	Fleiss’ kappa = 0.800
Cold et al. 26	2024	Denmark	3	FIT-positive colonoscopies, 1,405 colonoscopies.	17 experienced endoscopists	PDR	Agreement between AI and expert rating	Equal	Pearson’s correlation coefficient = -.42, *P* < 0.001.
Mean (range) or summary	2021 (2015–2024)	Asia: 5 studies Europe: 2 studies North America: 1 study	2.1 (1–5) hospitals.	695 (50–2,000) colonoscopies.	7.8 (4–17) endoscopists.	Agreement between AI and expert rating: 3 studies. AUC: 2 studies. ADR: 1 study AMR: 1 study PDR: 1 study	Agreement between AI and expert rating: 5 studies PDR: 2 studies Improvement by lavage: 1 study NA: 1 study	Equal: 6 studies Superior: 2 studies	Fleiss’ kappa: 3 studies Accuracy: 2 studies Interclass correlation coefficient: 1 study Chi square test: 1 study. Pearson’s correlation coefficient: 1 study
All studies compared agreement between their AI and expert rating, but used very heterogenous designs and reporting metrics to do so, which is explained in the last column of this table: “Reporting used for Comparison”. Therefore, a meta-analysis was not applicable for this systematic review, because only three studies (two using accuracy and one using Chi square test) tested whether the AI was superior to expert BBPS rating in classifying BBPS images correctly. The other five studies used correlations test, which do not detect superiority. AI-BPS, artificial intelligence bowel preparation scale; AUC, area under the curve; ADR, adenoma detection rate; AMR, adenoma miss rate; BBPS, Boston Bowel Preparation Scale; NA, not applicable; PDR, polyp detection rate; PMR, polyp miss rate.									

Six studies concluded their AI-BPS to assess bowel preparation equally to expert BBPS ratings, and two studies to be superior 20 21 . One study did it by showing a significant difference in ADR between AI-qualified and AI-unqualified bowel preparation evaluations that were insignificant based on expert BBPS ratings 21 . The other study did it by evaluating colonoscopy images with BBPS scores based on expert consensus, with the AI showing a higher accuracy in classifying those images than another group of expert raters.

Three studies were able to evaluate their AI-BPS according to established colonoscopy performance metrics: ADR 25 , AMR 21 , or PDR 26 .

### Development and performance of the AI-BPS systems

Six studies used expert BBPS ratings to train their AI-BPS ( Table 2 and Table 3 ). The two remaining studies utilized a pixel index to estimate bowel preparation, training their AI-BPS based on the ratio of pixels identified as feces to pixels identified as mucosa 19 26 . The studies were very heterogeneous in reporting performance of their outcome measures, with accuracy being the most frequently reported measure for internal validation by four studies (mean: 0.85; range: 0.59–0.94) and external validation by five studies (mean: 0.90; range: 0.84–0.95). Only three studies reported sensitivity and specificity for their internal and external validation.

**Table:** **Table 2** Development and validation of the AI-BPS systems.

Study	Name of AI system	Use of qualifier*	Description of system output	Method to train AI	Procedures for training of AI	Procedures for testing of AI (internal)	Procedures for testing of AI (external - images)	Procedures for testing of AI (external - video)	Output
Rosa-Rizzotto et al. 19	CCSP (Clean Colon Software Program)	No	Fecal mucosal index based on pixel coloring	Dupuis-Rosa-Rizzotto Index (Fecal/Mucosal ratio)	50 colonoscopies	NA	NA	50 colonoscopies rated by 4 expert endoscopists	BBPS score
Zhou et al. 20	ENDOANGEL (precursor for e-BBPS)	Yes	BBPS score	BBPS rating of images by 5 experienced endoscopists	4,764 images from > 2,000 colonoscopies	592 images	120 colonoscopy images evaluated by 3 expert endoscopists	20 colonoscopies rated by 5 expert endoscopists	BBPS classification of 30-second video segment
Zhou et al. 21	e-BBPS	Yes	Range 0–20 indicating the proportion of images with a BBPS score ≤ 1	BBPS rating of images by 5 experienced endoscopists	4,764 images from > 2,000 colonoscopies	500 images	1,600 images evaluated by 3 expert endoscopists	120 colonoscopies rated by 3 expert endoscopists	e-BBPS score of image or full colonoscopy video
Lee et al. 22	AI-BBPS	Yes	Adequate (AI-BBPS > 1) or inadequate (BBPS ≤ 1)	BBPS rating of images by 1 experienced endoscopists	26,950 images from 200 colonoscopies	10 colonoscopies	NA	252 ten-second colonoscopy segments from 36 colonoscopies rated by 2 expert endoscopists	BBPS score
Feng et al. 23	ViENDO	Yes	BBPS score	BBPS rating of 3 second video segments by two expert endoscopists	160 colonoscopies	40 colonoscopies	NA	48 colonoscopies rated by 6 endoscopists	BBPS score
Lee et al. 24	NA	No	BBPS score	BBPS rating of images from 3 experienced endoscopists	2,361 images from 693 colonoscopies	113 ten-second video segments from 14 colonoscopies	NA	225 colonoscopies rated by 3 endoscopists	BBPS score
Yao et al. 25	e-BBPS	Yes	Range 0–20 indicating proportion of images with a BBPS score ≤ 1	BBPS rating of images by 5 experienced endoscopists	4,764 images from > 2000 colonoscopies	500 images	1,600 images evaluated by 3 expert endoscopists	393 colonoscopies immediately rated by performing endoscopist	e-BBPS score
Cold et al. 26	OSABPS	No	Continous score with a threshold of 0.09 between adequate and inadequate cleansing	Fecal/mucosal ratio	50,000 images from 20 colonoscopies	1,405 colonoscopies	5,525 images with consensus from expert endoscopists	NA	OSABPS score
Mean (range) or summary	NA	Yes: 5 studies No: 3 studies	NA	Expert BBPS ratings: 6 studies Fecal/mucosal ratio: 2 studies	Images: 15600 (2,361–50,000) Colonoscopies: 791 (20–2,000)	Images: 531 (500–592) Colonoscopies: 367 (10–1,405)	Images: 2,211 (120–5,525)	Colonoscopies: 127 (20–393)	Automated BBPS score: 5 studies Different score: 3 studies (e-BBPS and OSABPS).
*Use of qualifier to distinguish between qualified and unqualified images for BBPS rating. AI, artificial intelligence; AI-BPS, artificial intelligence bowel preparation scale; BBPS, Boston Bowel Preparation Scale; NA, not applicable.									

**Table:** **Table 3** Performance measures for the AI-BPS systems.

Study	Accuracy (internal)*	Sensitivity*	Specificity*	AUC*	PPV*	NPV*	Accuracy (external) ^†^	Sensitivity ^†^	Specificity ^†^	AUC ^†^	PPV ^†^	NPV ^†^
Rosa-Rizzotto et al. 19	NA	NA	NA	NA	NA	NA	NA	NA	NA	NA	NA	NA
Zhou et al. 20	.92	NA	NA	NA	NA	NA	.93	NA	NA	NA	NA	NA
Zhou et al. 21	.59	NA	NA	NA	NA	NA	.94	NA	NA	NA	NA	NA
Lee et al. 22	NA	1.00	NA	NA	NA	NA	.85	.81	.87	.92	NA	NA
Feng et al. 23	.94	.84	100	.98	NA	NA	.92	.92	.92	NA	NA	NA
Lee et al. 24	.94	NA	NA	.939	NA	NA	.95	NA	NA	.98	NA	NA
Yao et al. 25	NA	NA	NA	NA	NA	NA	NA	NA	NA	NA	NA	NA
Cold et al. 26	NA	.97	.60	NA	.19	.99	NA	NA	NA	NA	NA	NA
Mean (range)	.85 (.59-.94)	.94 (.59-.94)	.8 (.6–1.0)	.96 (.59-.94)	.19 (.59-.94)	.99 (.99)	.92 (.85–95)	.87 (.81-.92)	.90 (.87-.92)	.95 (.92-.98)	NA	NA
*Internal validation. ^†^ External validation. AI-BPS, artificial intelligence bowel preparation scale;AUC, area under the curve; NA, not applied, indicates the heterogenous and often missing reporting conducted by the studies; NPV, negative predictive value; PPV, positive predictive value.												

### Quality assessment

MERSQI scores differed slightly (mean: 14.4; range: 13.5–16.0), reflecting similar study designs and approaches ( Table 3 ). The implementation study was the highest-scoring study, designed as a non-randomized two group-study (2 points) 25 with the rest being single-group observational assessment/development studies (1 point). Three studies scored the maximum 1.5 points for being from multiple institutions 19 23 26 , whereas the rest utilized only one institution (.5 points).

All studies used agreement between their AI-BPS and expert BBPS ratings as an outcome measure (1.5 points), with three studies also using agreement between their AI and ADR 25 , AMR 21 , or PDR 26 as outcome measures (3 points).

All studies scored points for Validity - Content by thoroughly justifying their rationale for conducting the assessment. All studies scored points for Validity - Relationship to other variables by comparing their AI-BPS result to either expert BBPS rating, ADR, AMR, or PDR. All studies except one 19 scored points for Validity - Internal structure, most frequently by using interrater reliability.

All studies scored maximum points for the following items: response rate (no studies reported on system failure, and therefore, all studies had an assumed response rate > 75%), type of data (all studies used automatic AI assessment corresponding to an objective measurement), appropriateness and complexity of analysis (all studies used appropriate analyses beyond descriptive statistics).

## Discussion

This is the first systematic review of AI-BPS systems. We identified eight studies that used different approaches to test their AI-BPS.

### Performance of AI-BPS: Expert agreement, ADR, AMR, and PDR

All studies compared agreement of their AIs and expert BBPS ratings ( Table 1 ). Conflicting results have been shown on whether BBPS can be used to correlate with ADR or AMR 10 11 12 32 . AI-BPS could thereby address shortcomings of expert BBPS ratings by correlating them with these established performance measures in colonoscopy. Only three studies achieved this: Zhou et al. 21 with ADR, Yao et al. 25 with AMR, and Cold et al. 26 with PDR.

Zhou et al. 21 established a correlation between ADR and their AI-BPS, called e-BBPS. e-BBPS classifies each frame of a colonoscopy video as either inadequately (BBPS = 0–1) or adequately cleansed (BBPS = 2–3). e-BBPS then calculates a total score ranging from 0–20 for the entire colonoscopy. Each point indicates a 5% interval of the frequency of images classified as inadequately cleansed; an e-BBPS of 3 equals 15% of images classified as inadequately cleansed, e-BBPS of 4 equals 20%, and so on. Therefore, the score is based on a dichotomous measure (inadequately or adequately cleansed) and does not utilize the full scale of the BBPS. AI systems can provide more granular assessments than human ratings, as highlighted by the two studies that used pixel counts of feces and mucosa to assess bowel preparation 19 26 . However, despite this limitation, Zhou et al. 21 were able to correlate e-BBPS to ADR, setting a threshold e-BBPS ≤ 3 as adequately cleansed, defined by the performance target of ADRs above 25% 33 . ADR steadily declined below 25% when e-BBPS rose to 4 and beyond. Thereby, e-BBPS could determine adequate cleansing based on ADR in contrast to major quality analysis programs using BBPS and endoscopist rating in their study 11 12 21 . However, the study was conducted as a single-center study, including only four endoscopists, and only reflected their cumulative ADR, decreasing the generalizability of the study 21 . Cold et al. 26 had greater generalizability by including 17 endoscopists from three hospitals and setting a threshold to distinguish adequate from inadequate bowel preparation, demonstrating a significant difference in PDR between the two. PDR has a lower correlation to PCCRC than ADR 2 . Cold et al. 26 highlighted use of PDR as a limitation of their study, suggesting that pathology reports should be retrieved to use ADR instead. ADR is the recommended gold standard for reporting colonoscopy quality 34 . Nonetheless, AMR has been more closely related to PCCRC 35 . AMR requires tandem colonoscopies, which are impractical in daily clinical practice 36 . However, Yao et al. 25 tested e-BBPS in a prospective tandem-colonoscopy study to determine if AMR was higher for AI-unqualified (e-BBPS > 3) than AI-qualified (e-BBPS ≤ 3) colonoscopies. Patients with AI-unqualified colonoscopies underwent re-cleansing before the second colonoscopy by the same endoscopist on the same day or the next day. AI-qualified colonoscopies were immediately reexamined by the same endoscopist. AI-unqualified colonoscopies had a higher AMR than AI-qualified colonoscopies (50.9% vs 20.8%, OR 0.25, *P* < 0.001) 25 . The AMR for the AI-qualified colonoscopies was similar to other studies, because a meta-analysis of 43 publications with more than 15,000 tandem colonoscopies showed a mean AMR of 26% 16 . Because insufficient bowel preparation is the primary cause of missed adenomas 5 6 , it is no surprise that the AMR was higher for the AI-unqualified colonoscopies, and the authors were able to reaffirm this sentiment in their study. In addition, five experts reviewed the recordings to identify adenomas missed due to insufficient bowel preparation, missed visible adenomas, and unexposed missed adenomas due to suboptimal mucosal exposure 25 . Two other types of AI systems have been developed to improve detection of adenomas. CADe can detect visible adenomas 13 and CAQ can help expose more of the mucosa 14 . AI-BPS, CADe, and CAQ systems could address the three reasons for hindered adenoma detection highlighted by Yao et al. 25 , demonstrating how AI could be a potential solution for increasing ADR for underperforming endoscopists. Another study has shown additive effects when combining CADe with CAQ 37 , and future studies should examine if AI-BPS can work in combination with CADe or CAQ. Unfortunately, the e-BBPS system is not commercial, which hinders its widespread distribution and testing. However, the study by Cold et al. 26 launched an open-source AI-BPS to address this issue.

### Development and performance measures of AI-BPS

One study provided the only open-source AI-BPS 26 . Because it is the most recent publication, no other research group has externally validated or implemented it yet. This is quite important, as it was the only study that did not use colonoscopy video segments but used solely images for external validation 26 ( Table 2 ). BBPS is intended to be applied to a whole segment of the colon, and solely external validation on images is a limitation. An average drop in performance of 6% is observed when externally testing AI in radiology 38 . Unfortunately, reporting of the eight included studies was very heterogenous and recommended performance measures were often missing 39 ( Table 3 ). The most frequent reporting measure was accuracy for internal validation reported in four studies (mean: 0.85, range: 0.59–0.94) and external validation by five studies (mean: 0.90; range: 0.84–0.95). Of the four studies reporting accuracy for both internal and external validation, accuracy increased by 11% (from 84% to 93%). However, an increase in performance at external validation is not uncommon 38 . It can arise from differences in dataset characteristics, such as image quality 40 . The Quality Assessment of AI studies in Diagnostic Endoscopy (QUAIDE) has developed a checklist to improve design and reporting 41 and other recommended reporting metrics regarding AI in endoscopy exist 39 . We highly recommend future studies follow these guidelines to enable comparison of performance via standardized reporting. For example, only three studies reported sensitivity and specificity for their internal and external validation, which should have been reported for all studies according to the guidelines 39 41 . The feces-mucosal ratio provides a more granular assessment, whereas expert BBPS ratings are a standardized and widely used method for evaluating bowel preparation. Due to the heterogeneous reporting and small number of included studies, this systematic review cannot make suggestions on which approach to use prospectively.

### Quality assessment

Methodological quality was high (MERSQI, mean = 14.4, range 13.5–16) ( Table 4 ). The high scores can, in part, be attributed to maximum scoring in Response Rate and Type of Data, which are intrinsic in studies developing AI 14 . All studies also scored maximum points for Validity - Relationship to other variables, which is the most frequent source of collected validity evidence 42 . We recommend doing so by correlating with patient-related outcome measures, preferably ADR or AMR, and not just expert BBPS ratings. AI-BPS are assessment systems, and their development should follow the recommendation of using a validity framework when developing them 42 . Using a validity framework can help researchers design better studies and practitioners to interpret results more effectively 42 . Only one study did not score points for Validity - Internal structure *,* which was most frequently assessed by interrater reliability. When training an AI based on expert rating, it is important that agreement is high, because the AI will amplify the bias it is trained on 43 44 45 . To limit the bias of human rating, all except one of the included studies used only images for which experts agreed on the BBPS rating to train the AI. Lee et al. 22 trained their AI using ratings of just one experienced endoscopist, making the AI a direct result of that endoscopist’s potentially biased rating, which cannot be recommended. When training AIs on human ratings, several raters should be included, and only images with consensus should be used. The internal structure can thereby be assessed by interrater variability.

**Table:** **Table 4** MERSQI-score.

Study	Study design (3)	Sampling (institutions) (1.5)	Sampling (response rate) (1.5)	Type of data (3)	Validity (internal structure) (1)	Validity (content) (1)	Validity (relationships to other variables) (1)	Data analysis (appropriateness) (1)	Data analysis (complexity) (2)	Outcomes (3)	Total (18)
Rosa-Riz. et al. 19	1	1.5	1.5	3	0	1	1	1	2	1.5	13.5
Zhou et al. 20	1	.5	1.5	3	1	1	1	1	2	1.5	13.5
Zhou et al. 21	1	.5	1.5	3	1	1	1	1	2	3	15
Lee et al. 22	1	.5	1.5	3	1	1	1	1	2	1.5	13.5
Feng et al. 23	1	1.5	1.5	3	1	1	1	1	2	1.5	14.5
Lee et al. 24	1	.5	1.5	3	1	1	1	1	2	1.5	13.5
Yao et al. 25	2	.5	1.5	3	1	1	1	1	2	3	16
Cold et al. 26	1	1.5	1.5	3	1	1	1	1	2	3	16
Mean (range)	1.1 (1–2)	.9 (.5–1.5)	1.5 (1.5)	3 (3)	.9 (0–1)	1 (1)	1 (1)	1 (1)	2 (2)	2.1 (1.5–3)	14.4 (13.5–16)
Numbers in parentheses below column headings are the maximum number of points for each category and the maximum total points. MERSQI, Medical Education Research Quality Instrument.											

### Limitations and future perspectives

Owing to the emerging nature of AI-BPS systems, this systematic review included only eight studies with diverse study designs and reporting metrics, and therefore, a meta-analysis was not possible. Future studies should, therefore, follow recommended reporting guidelines 39 . Several limitations of the included studies have been highlighted throughout the manuscript, but we wish to highlight one specifically, because it reflects the importance of using recommended reporting guidelines. Zhou et al. 21 set a performance target above 25% ADR based on a recommendation from the American Society for Gastrointestinal Endoscopy and European Society of Gastrointestinal Endoscopy 33 46 . However, the patient population between the recommendations (screening colonoscopies for patients aged ≥ 50 years) and the study patients aged ≥ 18 years) differed. Comparing ADR across patient populations should be done with the utmost caution 34 , and to use this performance target, the patient population should match the recommendation. This reflects the heterogeneous reporting of the included studies, and we highly emphasize using the most recent recommendations for developing AI and reporting ADR 34 39 41 46 47 . This review was not registered with open databases such as PROSPERO, which could have enabled others to conduct a similar review simultaneously.

e-BBPS correlated with ADR and AMR but did not estimate where in the colon the adenomas were missed 21 25 . Zhou et al. 20 were able to provide a bowel preparation score for every 30-second interval. Implementing such a system could increase ADR by alerting the endoscopist when an area is inadequately cleansed. When creating an assessment tool, its ability to enhance performance through feedback should be assessed in future studies, but unfortunately, only one study did so 25 . However, the study was limited by only dividing colonoscopies into adequately or inadequately cleansed, whereas adenomas might be missed in borderline cases. The withdrawal speedometer, which alarms the endoscopist when withdrawing too quickly, is the most studied CAQ system, with three studies showing an improvement in ADR when using it 14 . Similar studies are needed for AI-BPS, because AI-BPS seems promising in more accurately and objectively estimating bowel preparation to predict AMR and enhance ADR.

## Conclusions

This systematic review identified eight studies, with six AI-BPS that were trained based on expert BBPS ratings and two AI-BPS trained on a fecal-mucosal ratio. AI-BPS can standardize and outperform human rating of bowel preparation, by in some of the identified studies better correlating with expert BBPS ratings, AMR, ADR, and PDR. Future AI-BPS implementation studies following the most recent reporting recommendations are needed to alert the endoscopist when an area is insufficiently cleansed to increase ADR.

## Supplementary Material

Supplementary Material Zusatzmaterial

Supplementary Material Zusatzmaterial

Supplementary Material Zusatzmaterial
