# Supplementary material for: Bowel preparation assessment using artificial intelligence: Systematic review
Source: Endosc Int Open. 2025 Jul 1;13:a26256327. doi: 10.1055/a-2625-6327 (PMC12223940; doi:10.1055/a-2625-6327)
Supplement: Supplementary file 2 — Supplementary Material [file 10-1055-a-2625-6327_26299186.pdf]

**Supplementary material 2** Searches were conducted on August 15, 2024 and November 21, 2024, identifying 1,445 studies after removal of duplicates.

PubMed: 903 hits, November 21, 2024

(("Colonoscopy"[MeSH Terms] OR "colonoscopes"[MeSH Terms] OR "colonoscop*"[Text Word]) AND ("bowel preparation*"[Text Word] OR "bowel clea*"[Text Word] OR "BBPS"[Text Word] OR "aronchick scale*"[Text Word] OR "OBPS"[Text Word] OR "harefield cleansing scale*"[Text Word] OR "CBPS"[Text Word] OR "preparation scale*"[Text Word] OR "Cathartics"[MeSH Terms] OR "cathartic*"[Text Word]) AND ("valid*"[Text Word] OR "Validation Studies as Topic"[MeSH Terms] OR "certificat*"[Text Word] OR "Certification"[MeSH Terms] OR "goals"[MeSH Terms] OR "goal*"[Text Word] OR "objective*"[Text Word] OR "automatable*"[Text Word] OR "automatic*"[Text Word] OR "automatism"[MeSH Terms] OR "automatism*"[Text Word] OR "automatiz*"[Text Word] OR "unbias*"[Text Word] OR "artificial intelligence*"[Text Word] OR "artificial intelligence"[MeSH Terms] OR "computerised*"[Text Word] OR "computerized*"[Text Word] OR "ai based*"[Text Word] OR "ai assistance*"[Text Word] OR "deep lear*"[Text Word] OR "deep learning"[MeSH Terms] OR "software validation*"[Text Word] OR "Software Validation"[MeSH Terms] OR "machine lear*"[Text Word] OR "DNN"[Text Word] OR "neural network*"[Text Word] OR "CNN"[Text Word] OR "convolutional network*"[Text Word]) AND "english"[Language]) AND (english[Filter])

Scopus: 989 hits, November 21, 2024

Search: Article title, Abstract, Keywords:
( ( "Colonoscopy" OR "colonoscopes" OR "Colonoscop*" ) AND ( ( "bowel preparation*" OR "bowel clea*" OR "BBPS" OR "Aronchick scale*" OR "OBPS" OR "Harefield Cleansing Scale*" OR "CBPS" OR "Preparation Scale*" OR "Cathartics" OR "Cathartic*" ) ) ) AND ( ( "valid*" OR "Validation Studies as Topic" OR "certificat*" OR "Certification" OR "goals" OR "goal*" OR "objective*" OR "automatable" OR "automatic*" OR "automatism" OR "automatism*" OR "automatiz*" OR "unbiased" OR "artificial intelligence" OR "artificial intelligence" OR "computerised" OR "computerized" OR "AI based" OR "AI assistance" OR "deep lear*" OR "deep learning" OR "Software Validation*" OR "Software Validation" OR "Machine learning" OR "DNN" OR "Neural network" OR "CNN" OR "Convolutional network" ) ) Limit to English,
