# Supplementary material for: Bowel preparation assessment using artificial intelligence: Systematic review
Source: Endosc Int Open. 2025 Jul 1;13:a26256327. doi: 10.1055/a-2625-6327 (PMC12223940; doi:10.1055/a-2625-6327)
Supplement: Supplementary file 3 — Supplementary Material [file 10-1055-a-2625-6327_26299188.pdf]

Supplementary Fig. 1 EMBASE search.

Search History (20)

View Saved

#	Searches	Results	Type	Actions	Annotations
1	exp colonoscopy/	113691	Advanced	Display Results More	
2	(colonoscopy or colonoscope or colonoscopist or colonoscopic*).mp. [mp=title, abstract, heading word, drug trade name, original title, device manufacturer, drug manufacturer, device trade name, keyword heading word, floating subheading word, candidate term word]	123885	Advanced	Display Results More	
3	1 or 2	125081	Advanced	Display Results More	
4	exp cathartics/	199987	Advanced	Display Results More	
5	(bowel preparation* or bowel clea* or BBPS or Aronchick scale* or OBPS or Harefield Cleansing Scale* or CBPS or preparation scale* or cathartic*).mp. [mp=title, abstract, heading word, drug trade name, original title, device manufacturer, drug manufacturer, device trade name, keyword heading word, floating subheading word, candidate term word]	11730	Advanced	Display Results More	
6	4 or 5	208900	Advanced	Display Results More	
7	exp artificial intelligence/	120359	Advanced	Display Results More	
8	exp deep learning/	67561	Advanced	Display Results More	
9	exp software validation/	407	Advanced	Display Results More	
10	exp Validation Studies as Topic/	115917	Advanced	Display Results More	
11	exp certification/	23694	Advanced	Display Results More	
12	exp goals/	256738	Advanced	Display Results More	
13	exp automation/	2278	Advanced	Display Results More	
14	(valid* or certificat* or goal* or objective* or automatable* or automatic* or automation or automatiz* or unbiased or artificial intelligence or computerised or computerized or ai based or ai assistance or deep lear* or software validation* or machine lear* or DNN or neural network or CNN or convolutional network).mp. [mp=title, abstract, heading word, drug trade name, original title, device manufacturer, drug manufacturer, device trade name, keyword heading word, floating subheading word, candidate term word]	1277971	Advanced	Display Results More	
15	7 or 8 or 9 or 10 or 11 or 12 or 13 or 14	1467847	Advanced	Display Results More	
16	3 and 6 and 15	2372	Advanced	Display Results More	
17	limit 16 to conference abstracts	1145	Advanced	Display Results More	
18	16 not 17	1227	Advanced	Display Results More	
19	18	1227	Advanced	Display Results More	
20	limit 19 to english language	1154	Advanced	Display Results More	
